# Plasma synthesis of rhenium nanoparticles as an efficient alternative to platinum nanoparticles for nitroaromatic compound hydrogenations

**DOI:** 10.1038/s41598-025-25733-7

**Published:** 2025-11-25

**Authors:** Piotr Cyganowski, Dominik Terefinko, Mujahid Ameen Khan, Agata Motyka-Pomagruk, Mateusz M. Marzec, Sebastian Arabasz, Krystian Sokolowski, Pawel Pohl, Andrzej Bernasik, Aleksandra Goleniewska, Piotr Jamroz, Anna Dzimitrowicz

**Affiliations:** 1 Department of Process Engineering and Technology of Polymer and Carbon Materials, Wroclaw University of Science and Technology, 27 Wybrzeze St. Wyspianskiego, Wroclaw, 50-370 Poland; 2 Department of Analytical Chemistry and Chemical Metallurgy, Wroclaw University of Science and Technology, 27 Wybrzeze St. Wyspianskiego, Wroclaw, 50-370 Poland; 3 Laboratory of Plant Protection and Biotechnology, Intercollegiate Faculty of Biotechnology, University of Gdansk and Medical University of Gdansk, University of Gdansk, 58 Abrahama, Gdansk, 80-307 Poland; 4 Research & Development Laboratory, Intercollegiate Faculty of Biotechnology, University of Gdansk and Medical University of Gdansk, University of Gdansk, 20 Podwale Przedmiejskie, Gdansk, 80-824 Poland; 5 Academic Centre for Materials and Nanotechnology, AGH University of Krakow, Mickiewicza Av. 30, Kraków, 30-059 Poland; 6 Łukasiewicz Research Network - PORT Polish Center for Technology Development, 147 Stablowicka, Wrocław, 54-066 Poland; 7 Faculty of Physics and Applied Computer Science, AGH University of Krakow, 30 A. Mickiewicza, Kraków, 30-059 Poland

**Keywords:** Nitroarenes, Catalytic hydrogenation, Cold atmospheric pressure plasma, Seed germination, Pollution remediation, Catalyst synthesis, Homogeneous catalysis, Catalyst synthesis, Nanoparticles

## Abstract

Rhenium nanoparticles are an attractive alternative to noble metal-based approaches, which show a limited applicability. The catalytic potential and environmental impact of rhenium nanoparticles (ReNPs) were assessed in conjunction with those of platinum nanoparticles (PtNPs). Both of these nanomaterials were synthesized via low-cost pulse-modulated radiofrequency atmospheric pressure glow discharge (pm-rf-APGD). In this context, pm-rf-APGD was used for the first time to synthesize Re-based nanomaterials. The obtained nanoparticles were used as nanocatalysts for hydrogenation of nitroaromatic compounds. Subsequent characterization revealed high efficacy of rhenium nanoparticles in catalysing the reduction of nitroaromatic compounds, which reached up to 100% conversion yields at a rate constant *k*_*1*_ of 5.5 × 10^− 2^ min^− 1^. Although the *k*_*1*_ values obtained for rhenium nanoparticles were lower than those recorded for platinum nanoparticles, this research highlights the economic aspects and explores the possible optimization of catalytic systems. Some putatively disadvantageous environmental impact of rhenium nanoparticles and platinum nanoparticles was demonstrated by the exposure of *Raphanus sativus* var. *oleiferus* L. seeds to either rhenium nanoparticles or platinum nanoparticles that resulted in development of the shorter sprouts. Despite this negative environmental impact, the outcomes of this study highlight the potential of rhenium nanoparticles for chemical transformation and emphasize the importance of further insights into the economic and environmental aspects of their application and mitigation strategies.

**Supplementary Information:**

The online version contains supplementary material available at 10.1038/s41598-025-25733-7.

## Introduction

Currently, special attention is being given to the protection of the natural environment from unexpected air, water, and soil pollution^1–5^. In particular, water pollution is detrimental to human health. The United Nations (UN) reported that approximately 2.2 billion people lack access to drinkable water^6^ because of water pollution caused by industrial activities, urban development, and agricultural runoff^7–9^, resulting in the introduction of contaminants such as heavy metals, antibiotics, endocrine disruptors, and pathogens to the environment^7–9^.

In addition, society is concerned by the occurrence of nitroaromatic compounds (NACs) in the natural environment^10^. The widespread presence of these chemicals in industrial pipelines combined with emissions from combustion engines pose significant risks to human health and the environment, highlighting the urgent need for research into methods for their neutralization and removal^10–12^. Numerous methods for the neutralization of NACs involving different classes of advanced oxidation processes (AOPs)^13,14^, as well as the biological treatment of NACs, have been proposed^15^. Notably, hydrogenation processes constitute an attractive alternative to the above-mentioned procedures for NAC remediation as these approaches enable the reduction of NAC‒NO_2_ groups, resulting in the synthesis of aromatic amines (AAMs). Importantly, AAMs are recognized as important products for the large-scale pharmaceutical industry^16^.

Among various methods allowing for hydrogenation of NACs are non-catalysed^11,46^ and catalysed synthetic routes^17^. A particular focus has been attributed to the latter approach, because an extensive selection of metallic nanoparticles was recently reported to enable the reduction of NACs under mild conditions^18–20^. Among various nanoparticles (NPs), those based on noble metals are mostly desired owing to the well-documented efficiency of AuNPs, PtNPs, and PdNPs in supporting the reduction of NACs^20–26^. However, the extensive use of these nanomaterials suffer from major drawbacks, such as the high cost of these nanoparticles, which severely limits the potential applicability of these products^27,28^.

Recently emerging nanomaterials based on rhenium (Re) appear to be a valid alternative to the noble metal-based NPs. Re is 30 and 15 times less expensive than Au and the platinum group metals (PGMs), respectively^29^ (March 2024). In addition to a significant difference in price, compared with AuNPs and PGM NPs, Re has further advantages, making its nanomaterials even better catalysts in terms of supporting NAC hydrogenation processes^30–32^. ReNPs have a unique catalysis chemistry involving a wide spectrum of catalytically active Re species^33^, as well as the tendency of Re to form particularly small NPs, whose synthesis was recently increased even further by obtaining catalytically active Re atoms or clusters of atoms that were occasionally grouped into virtual/apparent NPs^32^. These properties make ReNPs not only tuneable, but also highly efficient catalysts and putatively a perfect fit for the processes of NAC remediation combined with the simultaneous synthesis of AAMs.

Although plasma-assisted synthesis of noble metal nanoparticles such as PtNPs has been described, to the best of our knowledge, no previous study has reported the fabrication of rhenium nanoparticles via pulse-modulated radio-frequency atmospheric pressure glow discharge (pm-rf-APGD). In this work, we demonstrate the first application of the ReNPs obtained by this method as catalysts for hydrogenation of nitroaromatic compounds. For direct comparison, PtNPs were synthesized under identical conditions, enabling us to benchmark the performance of Re against that of a well-established noble metal. Importantly, this approach not only validates the catalytic efficacy of plasma-synthesized ReNPs, but also reveals their distinctive structural and kinetic features, such as the formation of subnanometric clusters and an induction period in the hydrogenation pathway. Furthermore, this study integrates a preliminary environmental impact assessment, providing additional insight into the safe use of these nanomaterials. Collectively, our results establish ReNPs synthesized via pm-rf-APGD as a potential, cost-effective alternative to platinum-based catalysts and highlight the importance of further exploration of rhenium in applied catalysis.

## Results and discussion

### Apparent characteristics of rhenium and platinum nanoparticles

**Fig. 1:**
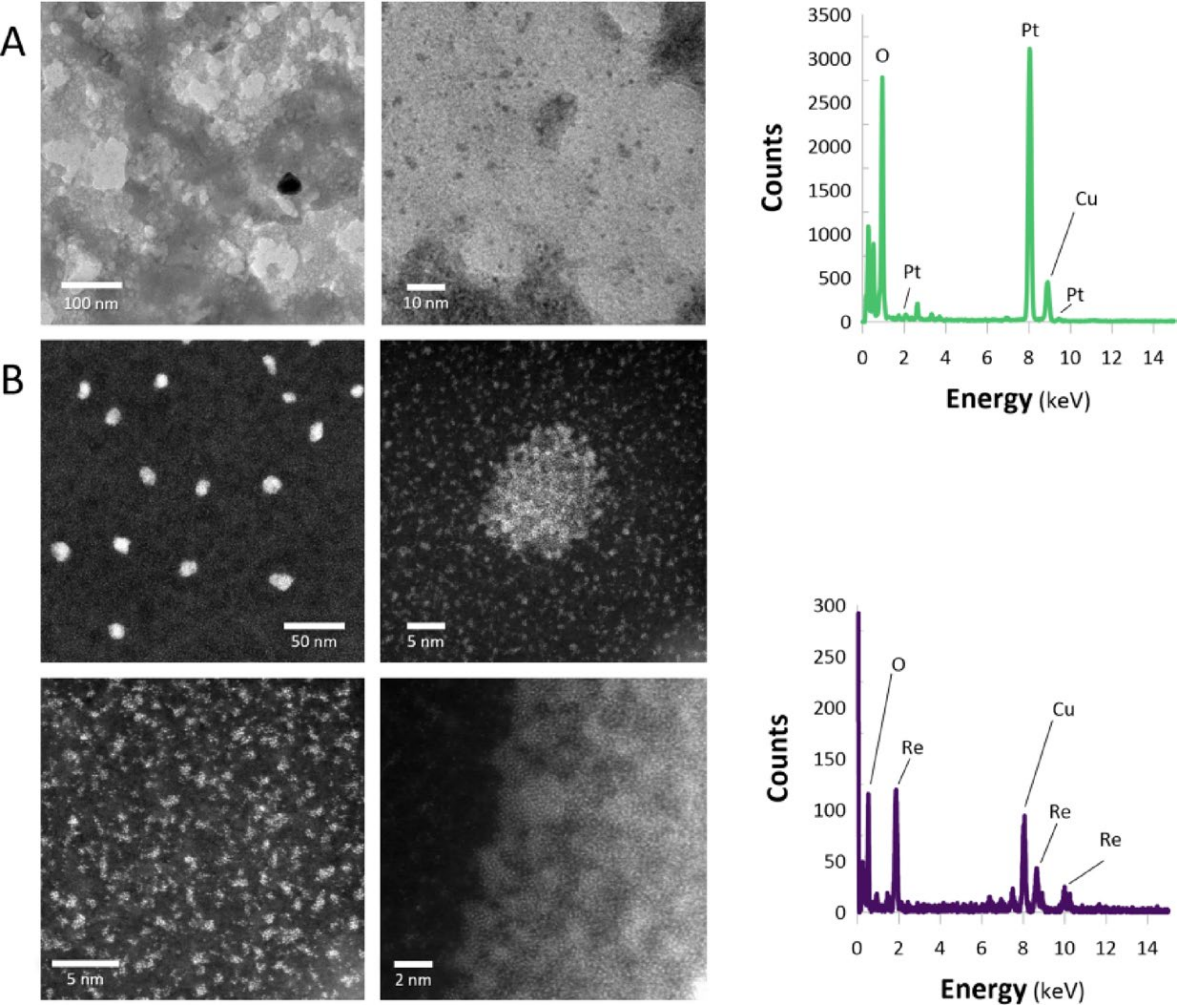
(**A**) TEM photomicrographs and EDS spectrum of the PtNPs. (**B**) STEM/HAAFD photomicrographs and EDS spectra of ReNPs and Re apparent NPs.

The synthesized ReNPs and PtNPs were first characterized via DLS, which involved determination of the NP hydrodynamic diameter (*D*_*H*_, nm) and the polydispersity index (*PdI*, %). The ReNPs had a *D*_*H*_ of 504.4 nm, and the PtNPs exhibited a *D*_*H*_ of 543.4 nm; the *PdI*s were 61 and 23%, respectively. Although Re is not accounted for by PGMs, it can form stable complexes similar to those of PGMs. Hence, similar *D*_*H*_ values attributed to the same mechanism of reduction were expected. On the basis of our previous experience^32^, the large difference in the *PdI*s may be assigned to the tendency of Re to form a variety of NPs, including particularly small ones (~ 1 nm and below). Hence, the samples of ReNPs and PtNPs were investigated by transmission electron microscopy (TEM), which, in the case of ReNPs, was aided by high resolution TEM (HRTEM) equipped with high angle annular dark field detector (HAADF) operating in scanning-transmission mode (STEM) and energy dispersive spectroscopy (EDS). The pictures captured during the analysis are shown in Fig. 1.

On the basis of the morphology of PtNPs shown in Fig. 1A, these nanostructures were highly dispersed and spherical in shape. The average size of the NPs equalled 2.13 ± 0.47 nm. In the case of the ReNPs, TEM was insufficient for observing the produced particles in spite of proofing the presence of Re (EDS spectrum, Fig. 1B), as there were no visible ReNPs. On the basis of our previous experience, we suspected that Re formed particularly small particles; thus, the analysis was performed with HRTEM instrument operating in STEM mode aided by HAADF. This decision was made because STEM mode provides generally better contrast of high-atomic-number objects (here, Re) on the carbon lattice than the TEM mode does.

STEM/HAADF analysis revealed that the reduction of Re(VII) ions produced numerous atom clusters (< 1 nm) (Fig. 1B). This structural behavior is unique to ReNPs and has not been reported for the plasma-synthesized noble metals. For the authors, it is debatable whether they can be referred to as nanoparticles or rather clusters of atoms. In many cases, individual atoms were also visible in the images. These atoms apparently tend to group, forming amorphous phases that seem to be “apparent nanoparticles” with larger sizes and unexpectedly regular shapes. Only at higher magnifications there were these agglomerates visible at the edges of the preparation. With the use of these agglomerates, the content of rhenium could have been confirmed (Fig. 1B). The relatively high oxygen content in the EDS spectrum indicated that an oxidized form of rhenium was likely present.

Unlike PtNPs, which formed well-defined spherical particles (Fig. 1A) suitable for statistical analysis, the Re-based structures appeared predominantly as subnanometric clusters and apparent nanoparticles. Because of their size and amorphous character, obtaining a robust statistical size distribution was not feasible. This limitation arose not from an insufficient resolution of the instrument, but rather from the properties of rhenium nanostructures, which tend to form atomically small clusters and irregular agglomerates.

### Identification of ReNPs

**Fig. 2:**
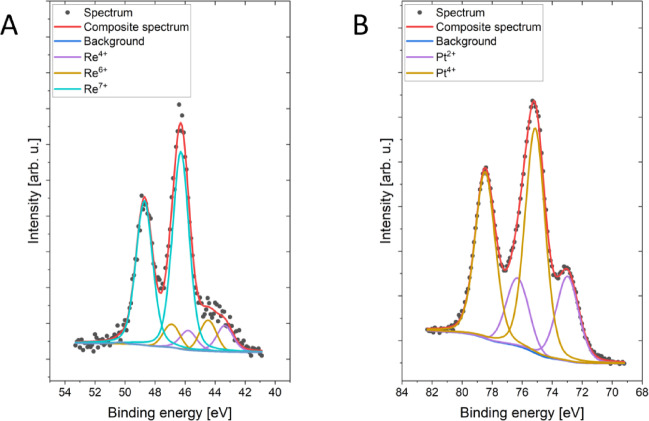
Re 4f and Pt 4f high-resolution XPS spectra of (**A**) ReNPs and (**B**) PtNPs.

The elemental composition and chemical state of the ReNPs and PtNPs were subsequently analysed by X-ray photoelectron spectroscopy (XPS). The survey scans as well as the high-resolution data used for the quantification of each oxidation state are shown in Fig. 2 and Figures S1–S4, while the surface composition (atomic %) determined by fitting the results of XPS is displayed in Table S1. The atomic concentrations of oxidation states for both types of NPs resulting from the fitting procedure are summarized in Table 1. The total concentration of rhenium was very low, but owing to the relatively high intensity of line 4f, the fitting was possible; thus, the presented concentrations were exceptionally rounded to the second decimal place.

**Table 1:** Surface composition (atomic %) determined by fitting the XPS data.

ReNPs	Re		
Binding energy [eV]	42.9	44.1	46.3
Sample/bonds	Re^4+^	Re^6+^	Re^7+^
Atomic %	0.05	0.09	0.56

**PtNPs**	**Pt**		
Binding energy [eV]	73.0	75.1	
Sample/bonds	Pt^2+^	Pt^4+^	
Atomic %	2.2	5.3	

The curve-fitting procedure of the Pt 4f region was performed with two doublets via a Pt 4f_7/2_–4f_5/2_ doublet separation of 3.3 eV, in which the first main 4f_7/2_ line at 73.0 eV indicates the presence of a Pt^2+^ oxidation state similar to that in PtO, and the second 4f_7/2_ line at 75.1 eV originates from Pt^4+^, as in PtO_2_^34,35^.

The Re 4f region was fitted with three doublets (doublet separation Re 4f_7/2_–f_5/2_ is equal to 2.4 eV), in which the first main line 4f_7/2_ at 42.9 eV indicates the presence of a Re^4+^ oxidation state similar to that in ReO_2_, the second line 4f_7/2_ centred at 44.1 eV is assigned to the Re^6+^ oxidation state similar to that in ReO_3_, and the third line 4f_7/2_ at 46.3 eV originates from the Re^7+^ oxidation state similar to that in Re_2_O_7_^34^.

In addition to the Re and Pt oxides, both NP samples contain some impurities, such as carbon, sodium, chlorine, nitrogen and silicon. For example, the C 1s spectra are similar to those of a typical carbonaceous contamination phase that covers the material surface because of exposure to air. Therefore, in the C 1s region, four lines are present at 285.0 eV from adventitious aliphatic C–C carbon and in the range of 286.6–288.9 eV from oxygen-containing species such as C–O and/or C = O and/or O = C–O. The Na 1s spectra were fitted with one line located at 1071.8 eV, which indicates the presence of sodium halides and/or oxides^34^. The Cl 2p spectra were fitted with one doublet (doublet separation 2p_3/2_–p_1/2_ is equal to 1.6 eV), with the main 2p_3/2_ line centred at 198.8 eV, which indicates the presence of Cl^-^ ions in chlorides^35^. The Si 2p spectra were fitted with three doublets (double separation p_3/2_–p_1/2_ is equal to 0.61 eV), in which the first 2p_3/2_ line at 98.9 eV indicates metallic silicon originating from the silicon wafer used as a substrate for the deposition of materials, the second 2p_3/2_ line at 101.8 eV points to the presence of Si–O bonds in silicone and/or siloxane compounds, and the third 2p_3/2_ line at 103.2 eV refers to the presence of silica^1^. The N 1s spectra were fitted with two lines; the first line at 400.2 eV corresponded to the presence of C–NH bonds, and the second line at 402.0 eV indicated the presence of NH_4_^+^ ions^34,36^. The O 1s spectra were fitted with three lines; the first line at 531.7 eV pointed to oxygen in metal oxides (O–Re, O–Pt) and/or O = C functional groups, the second line at 532.8 eV indicates the presence of O–C and/or O–Si bonds, and the last line at 533.9 eV unveiled –OH-type compounds and/or adsorbed water^37,38^.

### Catalytic hydrogenation of 4-NP over ReNPs and PtNPs

**Fig. 3:**
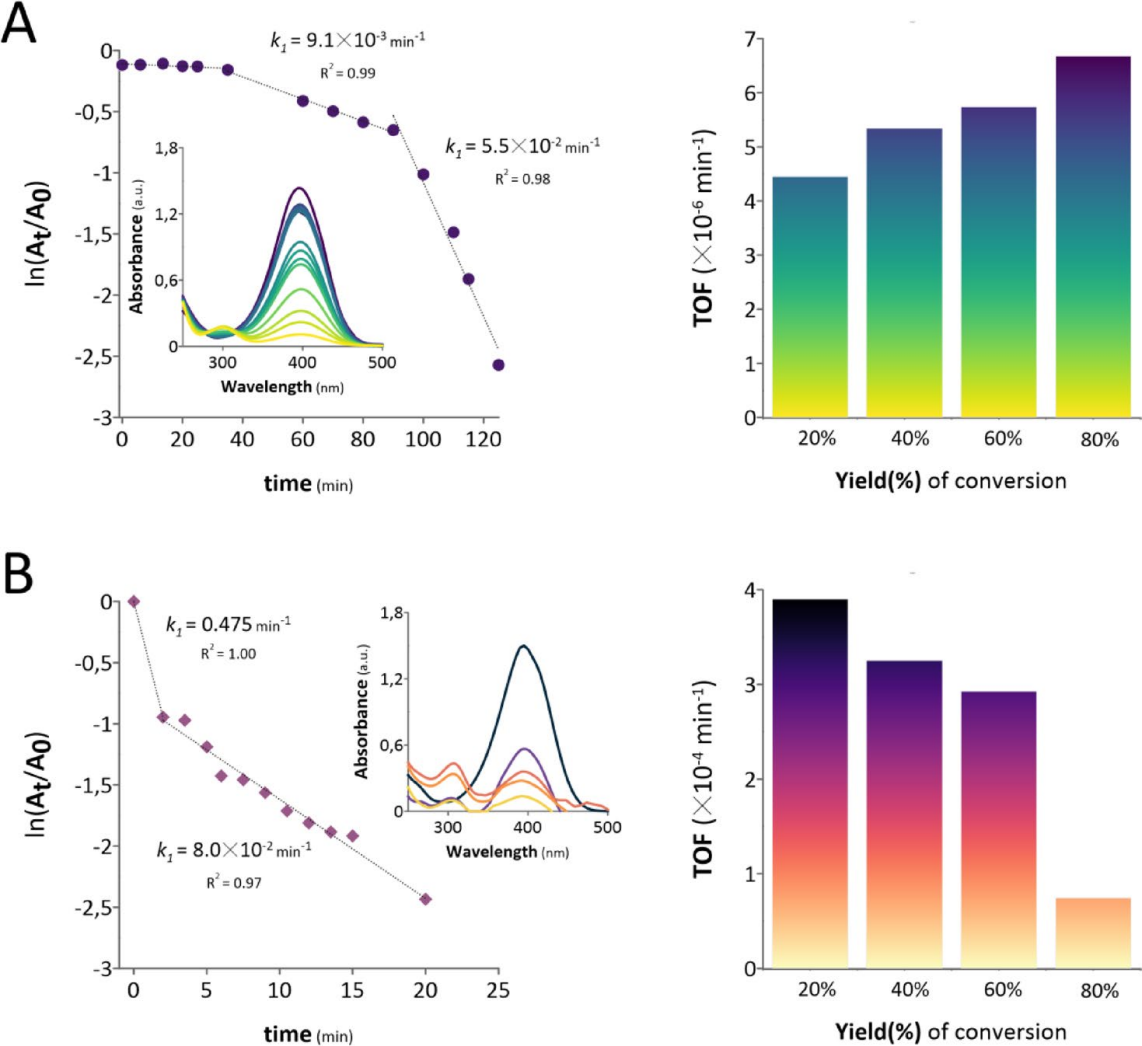
First-order kinetic plots, UV‒Vis spectra and TOF parameters of 4-NP catalytic reduction carried out over (**A**) ReNPs and (**B**) PtNPs.

ReNPs and PtNPs obtained in the pm-rf-APGD system were used as homogenous catalysts enabling the catalytic reduction of 4-nitrophenol (4-NP). The first-order kinetic plots and UV‒Vis spectra recorded for the catalytic process are shown in Fig. 3.

On the basis of the obtained data, both ReNPs and PtNPs successfully reduced 4-NP. The maximum yields (%) of hydrogenation were 93 and 91% for ReNPs and PtNPs, respectively. However, the reaction rate constants determined for these two catalysts differed significantly. As shown in Fig. 3, the values of *k*_*1*_ (min^− 1^) ranged between 9.1 × 10^− 3^ to 5.5 × 10^− 2^ min^− 1^ in the case of the ReNPs (Fig. 3A) and 8 × 10^− 2^ to 0.475 min^− 1^ regarding PtNPs (Fig. 3B). Furthermore, ReNPs had an induction period for 4-NP reduction, which resulted in negative reaction progress for more than 40 min. Moreover, the initial significant decrease in the 4-NP concentration occurred immediately after the catalyst was added to the system (Fig. 3). This decrease resulted in a significant difference between these catalysts in terms of the time needed to achieve the maximum 4-NP conversion. These values were 125 and 20 min for ReNPs and PtNPs, respectively. This finding may suggest that there was an additional process that might have occurred in the catalytic reduction of 4-NP over ReNPs. According to the recent work of Neal et al.^39^, the catalytic reduction of 4-NP in the presence of NaBH_4_ may have involved a side reaction, in which the synthesized 4-aminophenol (4-AP) can be reoxidized back into 4-NP after it is desorbed from the surface of a catalyst^39^. The produced 4-NP again reabsorbs onto the catalyst surface and is subjected to the reduction once again. These two processes are opposed to each other, and as a result, in spectrophotometric terms, the reaction may appear to be stalled. The side reaction occurs until O_2_ is present in the reaction mixture, and its occurrence ultimately fades as BH_4_^−^ ions scavenge O_2_, resulting in the formation of H_3_BO_3_, as proposed in the recent studies^39–41^. Hence, afterwards, the induction period ends, and the reduction in 4-NP advances.

In the present study, however, no induction period was observed in the case of PtNPs. This observation though does not mean that there was no side reaction of the 4-AP reoxidation described above, because there are numerous catalysts with similar mechanisms of operation^39–41^. The listed finding may suggest, however, that PtNPs may facilitate O_2_ scavenging mechanisms by catalysing O_2_ consumption by BH_4_^−^^39^, thus eliminating the induction period. This may mean that the PtNPs are better catalysts than the ReNPs. Furthermore, the reaction carried out with the use of ReNPs clearly involved a three-stage process (Fig. 3A), suggesting additional interactions occurring between the ReNPs and the reaction mixture. On the basis of our previous studies^42^, ReNPs are susceptible to interactions with O, and owing to the reactivity of Re at different oxidation states, the obtained species may be further reduced to other forms via NaBH_4_. Therefore, a possible explanation for the shape of the pseudo-first-order kinetics plot presented in Fig. 3A could be as follows: In the first stage of the reaction, there was an induction period. In the second stage, the reaction seemed to progress up to 48% 4-NP conversion. Afterwards, some ReNPs underwent transformation, as in the third stage, and the reaction progressed faster to complete at 93% 4-NP reduction. The drawn conclusion is further supported by the calculated TOF parameters (Fig. 3), which define the molar activity of a catalyst. The activity of ReNPs, unlike that of the PtNPs (Fig. 3B), gradually increased.

The above-described feature may be considered a major drawback of ReNPs in contrast to PtNPs. However, Re is more than 15-fold cheaper than Pt (data from March 2024)^29^, and still, ReNPs eventually resulted in almost complete conversion of 4-NP. Hence, there is a perspective for increasing the concentration of Re after catalyst preparation without exceeding the cost of the equivalent Pt-based catalyst. In our previous work, a 2000 mg L^−1^ Re(VII) ion solution was used in a direct current (dc) APGD system (here, it was 100 mg L^−1^ of Re(VII)). After this catalyst was applied, the resulting *k*_*1*_ value defined for 4-NP reduction was 160 min^−1,^ and no induction period was observed^43^. On the basis of the market price^29^, the application of these catalysts would be almost equally expensive as the use of the PtNPs in the present study. In this scenario, if the concentration of Re(VII) increases to 2000 mg L^−1^, the obtained *k*_*1*_ for this catalyst could have been 2000-fold greater than the *k*_*1*_ of the exactly the same-price PtNPs used in the current study. Although these are only theoretical considerations, they suggest that there is much space for optimization that might result in the fabrication of ReNPs that are not only equally effective as PtNPs, but also much less expensive.

To determine whether the ReNPs may be considered versatile catalysts, this unique nanomaterial was further used in the catalytic hydrogenation of other NACs, including 4-nitroaniline (4-NA), nitrobenzene (NB), 2,4-dinitrophenol (2,4-DNP), and 2,4,6-trinitrophenol (2,4,6-TNP). The collected data are summarized in Table 2.

**Table 2:**
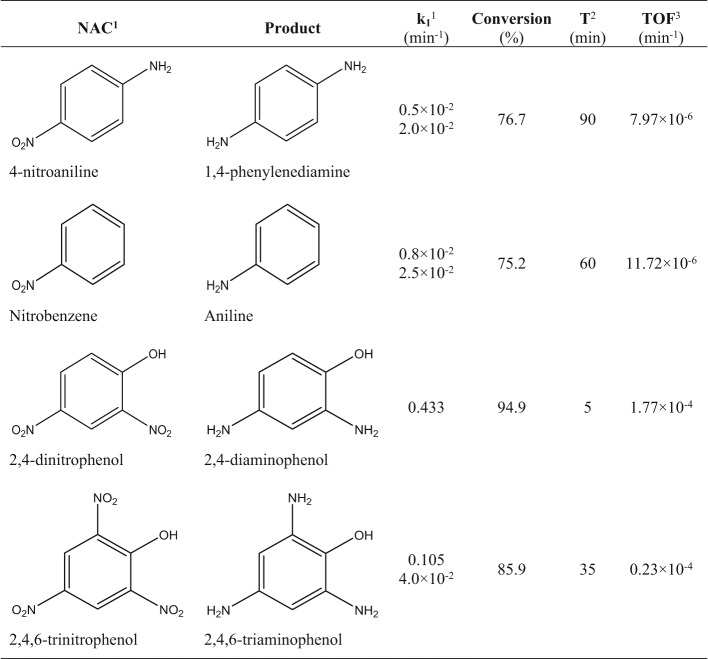
Catalytic hydrogenation of NACs over ReNPs.

^1^ first-order rate constant, ^2^ time needed to achieve maximum conversion, ^3^ turnover frequency parameter.

The displayed data confirm that the ReNPs enabled successful hydrogenation of 4-NA, NB, 2,4-DNP, and 2,4,6-TNP in addition to 4-NP. In most cases, the catalytic reaction was a two-stage process similar to 4-NP reduction. However, the reactions still occurred, and as discussed above, the cost of ReNPs is significantly lower than that of PtNPs. The first-order rate constants ranged from 0.5 × 10^− 2^ to 0.105 min^− 1^ in the case of 4-NA, NB and 2,4,6-TNP reduction, resulting in 76–86% yields of hydrogenation within 35–90 min. These results are consistent with the model reaction of 4-NP reduction that occurred in a similar manner.

Interestingly, catalytic hydrogenation of 2,4-DNP performed better, as a single-stage process characterized by a *k*_*1*_ value of 0.433 min^− 1^, and resulted in a 95% reduction within just 5 min. Similar observations can be made for hydrogenation of 2,4,6-TNP, which performed well, with a *k*_*1*_ of 0.105 min^− 1^ during the first stage of the process and ultimately slowed down in the second stage, but still outperformed the reduction of 4-NA and NB in addition to 4-NP (Table 2). This phenomenon may be linked to the structures of 2,4-DNP and 2,4,6-TNP. The presence of excessive electron withdrawing‒NO_2_ groups affects the aromatic ring in such a way that it is more electrophilic, and as a result, compared with the other NACs, the solutions of 2,4-DNP and 2,4,6-TNP tended to be characterized by a lower pH^44^. A lower pH facilitated the hydrolysis of NaBH_4_, and consequently, more reduction carriers (H_2_) were produced. This outcome further increased catalytic hydrogenation^45^. The differences in the progress of the catalytic reaction between 2,4-DNP and 2,4,6-TNP may be simply related to the number of ‒NO_2_ groups that these compounds contain. On the basis of our previous experience^32^, the number of ‒NO_2_ molecules to be reduced during a stated reaction causes a decrease in the molar activity of a catalyst. The previous observations did not differ from those of the present studies, because the calculated TOF parameters are consistent with the conclusions drawn above. Firstly, the molar activity of the ReNPs is greater for the catalytic hydrogenation of 2,4-DNP and 2,4,6-TNP than for that of the other NACs (effect of pH). Secondly, the presence of 3‒NO_2_ groups instead of 2‒NO_2_ groups resulted in a decreased activity of the ReNPs (Table 2).

Notably, the differences observed between ReNPs and PtNPs can be rationalized by considering both structural and electronic factors. TEM and STEM/HAADF (Fig. 1) analyses indicated that Re does not form conventional nanoparticles that are a few nanometres in size, but instead generates subnanometric clusters and apparent nanoparticles. These structures may have provided a higher degree of dispersion and exposed a greater density of catalytically active sites than did the more uniform and compact PtNPs. The morphology, unique to ReNPs synthesized by the plasma-based methods, is consistent with their ability to sustain high conversions despite the presence of an induction period. In addition, XPS analysis revealed that the surface of ReNPs contained a mixture of oxidation states, including Re(IV), Re(VI), and Re(VII) (Fig. 2; Table 1). The coexistence of these states creates multiple electron-transfer pathways that can facilitate the stepwise reduction of nitro groups to amines^46^. In contrast, the PtNPs were primarily present in the Pt(II) and Pt(IV) states, which may have provided narrower redox flexibility. It is possible that this redox diversity of Re contributes to the observed catalytic performance, particularly in the hydrogenation of substrates such as 2,4-DNP and 2,4,6-TNP, in which multiple nitro groups require successive electron transfer steps.

### Environmental impact of the ReNP and PtNP nanocatalysts

**Fig. 4:**
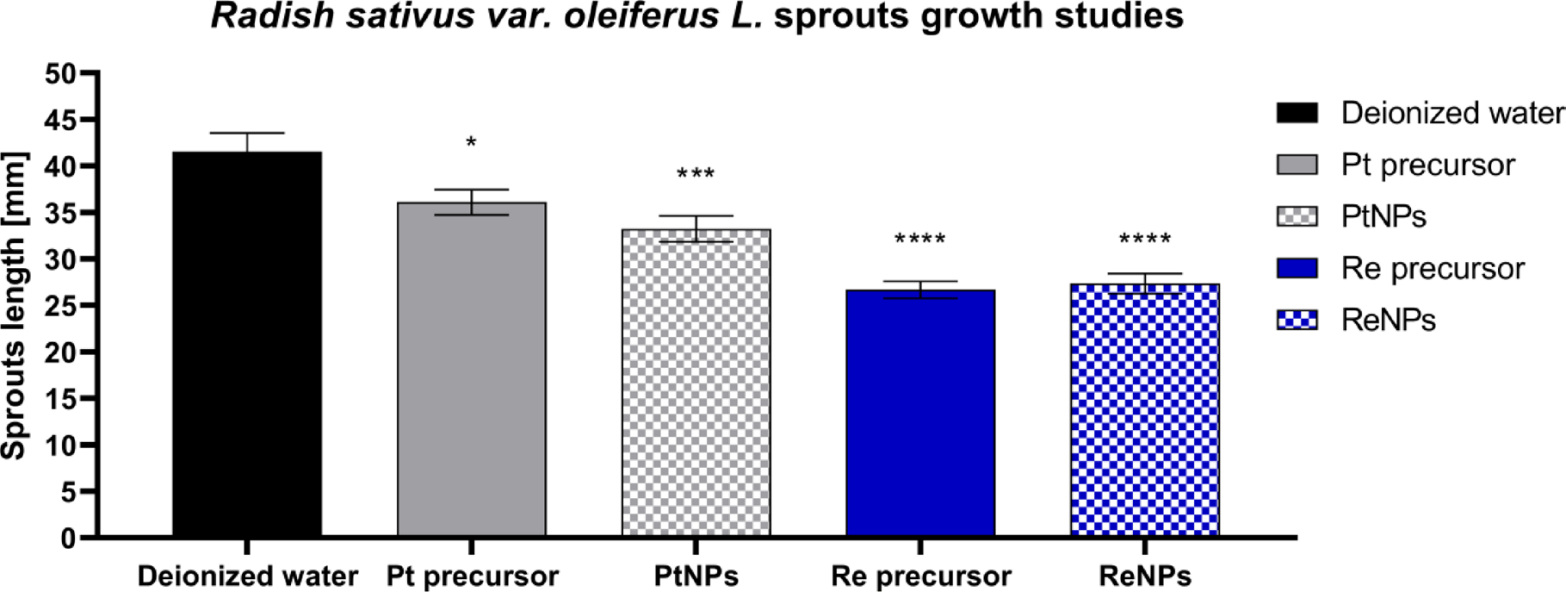
The lengths of *Raphanus sativus* L. var. *oleiferus* sprouts were measured 7 days after the plants were exposed to deionized water, Pt and Re ions at the concentration of 100 mg L^− 1^, and a dispersion of PtNPs or ReNPs. One-way ANOVA prior to Dunnett’s multiple comparisons test was applied to determine the statistical significance between deionized water and the corresponding solutions (* *p* < 0.05; *** *p* < 0.001; and **** *p* < 0.0001).

Owing to the beneficial properties of modern nanomaterials and the development of new routes for their use, their appearance in the natural environment is increasing; thus, the greatest concern should be focused on identifying ways to handle their dispersion, storage, and disposal^47^. From this perspective, experiments providing insight into the putative environmental impact of ReNP and PtNP nanocatalysts were performed to highlight the necessity of undertaking further security measures that should be considered after the application of ReNPs and PtNPs in catalytic hydrogenation processes. In terms of the lengths of sprouts of *Raphanus sativus* L. var. *oleiferus*, significant changes were observed between the groups treated with deionized water and those watered with ReNPs, PtNPs or the corresponding precursor solutions (Fig. 4).

Notably, both the analysed metallic precursor solutions significantly inhibited the growth of *R. sativus* sprouts, whereas the rhenium precursor had a stronger effect (**** *p* < 0.0001). Although compared with the corresponding precursor solutions, the exposure to PtNPs resulted in greater inhibition of early sprout growth (*** *p* < 0.001), the decrease in the mean sprout length for the group treated by ReNPs was comparable to that of the corresponding Re precursor (**** *p* < 0.0001). Nevertheless, the effect of exposure to Re on early plant growth was notable.

The results suggest that both ReNPs and PtNPs may have several detrimental environmental effects. The greater putative effect of ReNPs than of PtNPs may be associated with the reactivity of Re-based species, whereas Pt-based materials tend to be generally considered chemically inert. While the observed inhibitory effects of ReNPs on early plant growth and development are concerning, ReNPs have several advantageous characteristics that merit further attention.

## Conclusions

This study demonstrates for the first time the synthesis of rhenium nanoparticles (ReNPs) by pulse-modulated radio-frequency atmospheric pressure glow discharge (pm-rf-APGD) and their application as catalysts for nitroaromatic hydrogenation. While plasma-assisted routes for noble metal nanoparticles such as PtNPs are documented, the present work establishes ReNPs as a new class of plasma-fabricated catalysts.

Catalytic tests revealed that compared with PtNPs, ReNPs can achieve high conversions of nitroaromatic compounds, albeit of slower kinetics. Importantly, the direct side-by-side comparison reveals that despite having lower rate constants, ReNPs remain highly attractive due to their markedly lower cost and the distinctive mechanistic features observed, such as subnanometric cluster formation and three-stage kinetic profile. These insights provide a new understanding of the catalytic chemistry of rhenium, which differs fundamentally from that of Pt. Importantly, the synthesis route via pm-rf-APGD avoided the use of toxic reducing and capping agents. Despite the elimination of additional chemicals, early plant growth assessments revealed that both ReNPs and PtNPs had some detrimental environmental impacts, with ReNPs resulting in slightly greater impairment of this process. This observation was associated with the reactivity of the Re-based species. Nonetheless, the action of the ReNPs synthesized via the pm-rf-APGD method was comparable to that of the Re(VII) solutions. Despite the observed limitations, this work creates opportunities for further research into the optimization of ReNP synthesis, which may benefit from the significant difference in price between Re and noble metal-based solutions. ReNPs have the potential to be considered as new nanocatalysts for broader practical applications.

## Methods

### Reagents and solutions

To synthesize the ReNPs and PtNPs, two stock solutions were used. These solutions were prepared by dissolving either NH_4_ReO_4_ or H_2_PtCl_6_ in deionized water to obtain 500 mg L^− 1^ Re(VII) and Pt(VI) ions. Afterwards, the stock solutions were diluted to obtain working solutions of 100 mg of a stated metal per L. All of the reagents listed in this manuscript were acquired from Merck (branch Poland) and were of analytical grade or better. Deionized water was used throughout. For the ecotoxic studies, a 3% solution of hydrogen peroxide was prepared by diluting the obtained 30% solution 10 times. A 70% solution of ethanol was prepared by properly diluting its 96% solution. Both reagents were obtained from STANLAB (Poland). The *R. sativus L. var. oleiferus* seeds were kindly gifted by one of the Polish companies selling the seeds.

### Synthesis of metallic nanostructures

**Fig. 5:**
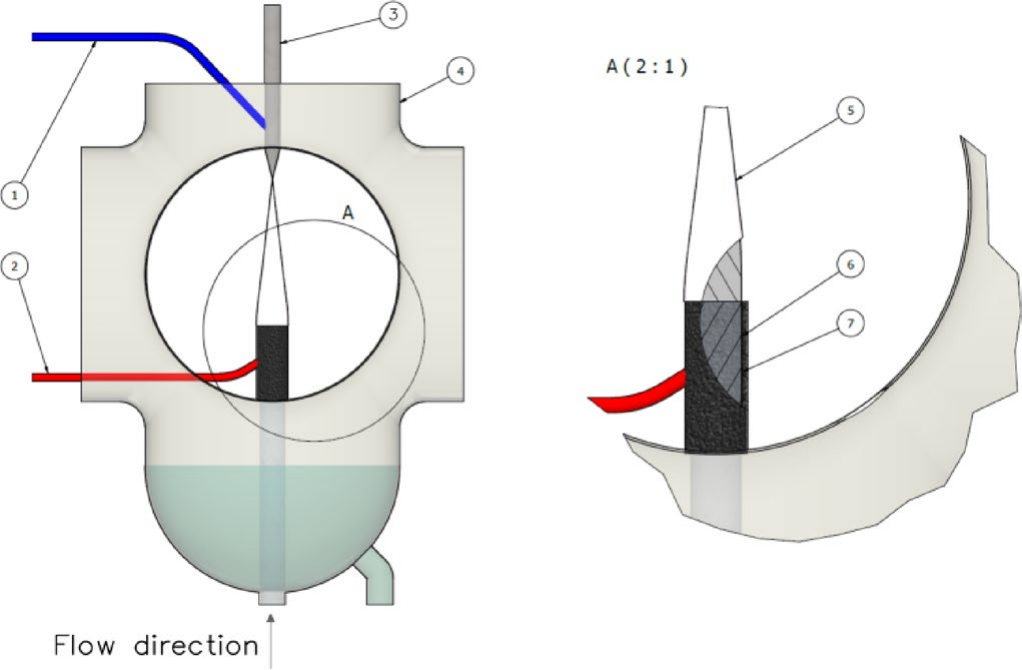
Pulse modulated radio frequency atmospheric pressure glow discharge (pm-rf-APGD) system applied as a CAPP source for the synthesis of ReNPs and PtNPs: (**1**,**2**) high-voltage wires, (**3**) tungsten electrode, (**4**) quartz chamber, (**5**) pm-rf-APGD, (**6**) graphite tube, and (**7**) quartz tube. The figure was prepared using Autodesk Inventor Professional 2025 software within the license granted to Wroclaw University of Science and Technology.

To produce ReNPs and PtNPs, a flow-through open-to-air cold atmospheric pressure plasma (CAPP) -based system was used. The elements of this system are shown in Fig. 5 and are operated as follows:

In this system, pm-rf-APGD was applied as a CAPP source and ignited between a pin-type metallic rod composed of tungsten (OD = 3.2 mm) and a continuously flowing PtNP or ReNP precursor solution (100 mg L^− 1^). The working solutions were introduced to the pm-rf-APGD by a two-channel peristaltic pump (Masterflex L/S, Core Palmer, USA) through a silicone tube and quartz-graphite tube (OD = 6.00 mm) at a flow rate of 2.0 mL min^− 1^. After the discharge chamber was reached, the Re(VII) and Pt(VI) ions were treated by pm-rf-APGD, and the resulting colloid solutions were gathered into glass vials for subsequent analyses. The current‒voltage characteristics of pm-rf-APGD were as follows: frequency of modulation of 1700 Hz and a duty cycle of 20%.

### Properties of the obtained nanoparticles

The granulometric properties of the ReNPs and PtNPs were estimated via dynamic light scattering (DLS), transmission electron microscopy (TEM) supported by energy dispersion X-ray scattering (EDS) and selected area electron diffraction (SAED).

With respect to the DLS measurements, approximately 3.0 mL of the analysed colloidal dispersion was poured into an optically homogenous square polypropylene cuvette and placed into the instrument (Litesizer 500, AntonPaar GmbH, Austria) to assess the size of the analysed NPs by number, volume, and intensity. The collected data were evaluated with Kaliope software (AntonPaar GmbH, Austria).

For TEM/EDS analysis, one drop of a colloidal suspension containing ReNPs or PtNPs was placed onto a Cu‒C grid (CF400-Cu-UL, Electron Microscopy Sciences, USA), moved into a quartz holder, and placed before evaporation inside a Tecnai G^2^ 20 X-TWIN instrument (Thermo Fisher, USA), which was equipped with an AZtecEnergy EDAX system. The morphology of the NPs was evaluated with FEI Tecnai™ G^2^ TEM software. Because of the tendency of Re to form particularly small structures, the ReNPs were additionally analysed via high-resolution transmission electron microscopy (HRTEM) aided by a selected area electron diffractometer, EDAX, and a high-angle annular dark-field (HAADF) detector in scanning transmission electron microscopy (STEM) mode (FEI TITAN^3^). In this case, approx. 5 µL of ReNPs was placed on Cu grids and then visualized under the (S)TEM Titan3 G2 60–300 microscope. The analyses were performed at an accelerating voltage of 300 kV and in STEM mode. The average size of the NPs based on the TEM analyses was calculated from measurements of at least 50 nanostructures with ImageJ software.

Quantitative measurements of the elements and their chemical states on the ReNP and PtNP surfaces were performed by a scanning X-ray photoelectron spectrometer (XPS) - a PHI 5000 VersaProbe^™^ II via a monochromatic Al Kα (1486.6 eV) focused X-ray beam on a 100 μm spot.

Phase identification of the ReNPs and PtNPs was carried out by determining the oxidation states of these metallic forms. For this purpose, XPS analyses were performed in a VersaProbeII scanning XPS system via monochromatic Al Kα (1486.6 eV) X-rays focused on a 100 μm spot. The photoelectron take-off angle was 45°, and the pass energy in the analyser was 117.50 eV (0.5 eV step) for survey scans and 46.95 eV (0.1 eV step) for high-resolution spectra. Dual-beam charge compensation with 7 eV Ar^+^ ions and 1 eV electrons was used to maintain a constant sample surface potential regardless of the sample conductivity. All XPS spectra were charge referenced to the unfunctionalized, saturated carbon (C–C) C1s peak at 285.0 eV. The operating pressure in the analytical chamber was less than 3 × 10^−9^ mbar. Deconvolution of the spectra was carried out via PHI MultiPak software (version 9.9.3). The spectrum background was subtracted via the Shirley method.

### Determination of the putative environmental impact of metallic nanoparticles

The evaluation of the effect of ReNPs and PtNPs on early plant growth, in this case conducted on *Raphanus sativus* L. var. *oleiferus*, was based on the modified protocol of Motyka-Pomagruk et al. (2021)^48^.

Firstly, the plant seeds were sterilized according to the following protocol. *R. sativus L. var. oleiferus *seeds were introduced into a plastic container and immersed for 30 s in 70% ethanol. After the ethanol was discarded, the seeds were washed in sterile deionized water for 60 s with occasional mixing. After removal of the deionized water, the oilseed radish seeds were subjected to 3% hydrogen peroxide for 300 s and subsequently shaken. Secondly, the 3% hydrogen peroxide solution was removed, and the seeds were subsequently washed with sterile deionized water four times; each incubation lasted for 60 s and included occasional mixing. The prepared surface sterilized *Raphanus sativus* L. var. *oleiferus* seeds were further used to evaluate early plant growth as follows.

On a standard ventilated Petri dish with an outer diameter of 93 mm, three sterile paper filter discs were placed to cover the bottom. Next, 1.50 mL of the analysed solution (ReNPs or PtNPs) was introduced into paper filters on a single Perti dish. As a negative control, sterilized deionized water was used, while in terms of the positive controls, the Pt and Re precursor solutions were included (100 mg L^− 1^). With sterile tweezer, nine *Raphanus sativus* L. var. *oleiferus* seeds were placed at equal distances on moistened paper filters on a single Petri dish. For each biological experiment, three Petri dishes with nine seeds on each were prepared. In total, three independent biological experiments involving nine Petri dishes with 81 seeds were conducted. These Petri dishes with oilseed radish seeds were incubated at 21 °C for 48 h. An additional aliquot of 1.50 mL of the proper solution was subsequently added to each Petri dish. The watering of the seeds was performed three times on days 1, 3, and 5 of the experiment. Lastly, 7 days after the experiment began, the sprouts were imaged with a scale for the length measurements. The data on the calculated lengths of the sprouts after 7 days of the experiment were processed with ImageJ software (version 1.54f; U.S. NIH). The results are presented as median values with SEM from three independent experiments with three technical repetitions. Graphical presentation and statistical analysis were performed via GraphPad Prism software (version 9.4.0; GraphPad Software, Boston). Statistical comparisons between sprout lengths were performed by comparing the results collected for the negative control (water) with those for the analysed solutions via one-way ANOVA with Dunnett’s multiple comparisons post hoc test.

### Catalytic hydrogenation of nitroarenes

The catalytic potential of ReNPs and PtNPs was assessed during the reduction of 4-NP. For this purpose, the reaction was carried out in the presence of NaBH_4_ as a reduction carrier. The change in the concentration of 4-NP over time was monitored using a UV‒Vis spectrophotometer (JASCO V-570, MD, USA). Firstly, 2.5 mL of 4-NP (0.1 mmol L^− 1^) was introduced into a quartz cuvette, and the absorbance spectrum (*λ*_*max*_ of 318 nm) was recorded. Secondly, 0.5 mL of NaBH_4_ (0.1 mol L^− 1^) was added to the cuvette, and the spectrum was recorded. At this point, the obtained spectrum showed an absorbance maximum at 400 nm, which is attributed to the formation of 4-nitrophenolate anions (O_2_N–Ar–O^−^). Lastly, 0.5 mL of ReNP or PtNP suspension was introduced into the cuvette, and the change in the absorbance at 400 nm was monitored at specific intervals by recording the spectra. The acquired initial absorbance (*A*_*o*_) at *λ*_*max*_ 400 nm and the absorbance at specific intervals (*A*_*t*_) at the same *λ*_*max*_ were used for pseudo-first order kinetic modelling by plotting *ln(A*_*t*_*/A*_*o*_*)* against the time (min) function. The slope value of the plot was determined as the pseudo-first-order rate constant (*k*_*1*_ min^− 1^). The molar-dependent activity of each catalyst was estimated via the turnover frequency (TOF, min^− 1^), defined as n_4 − NP_ × r × n_metal_^−1^ × t^− 1^, in which n_4 − NP_ and n_metal_ represent the number of moles of 4-NP and a metal (Re or Pt) at the start of the process, respectively, t is the processing time (min), and r is the reduction yield (%) at which the TOF is calculated.

ReNPs were further used in the catalytic hydrogenation of NB, 4-NA, 2,4-DNP, and 2,4,6-TNPEnergy Dispersive Spectroscopy. The procedure was exactly the same as that for the catalytic reduction of 4-NP, with the difference being that the absorbances at *λ*_*max*_ values of 275, 380, and 390 nm were monitored and used for the first-order kinetics.

## Supplementary Information

Below is the link to the electronic supplementary material.

Supplementary Material 1

## Data Availability

All the data associated with this manuscript are available at the following permanent identifier: (10.18150/WSWPQT).
