# Supplementary material for: Plasma synthesis of rhenium nanoparticles as an efficient alternative to platinum nanoparticles for nitroaromatic compound hydrogenations
Source: Sci Rep. 2025 Nov 25;15:41789. doi: 10.1038/s41598-025-25733-7 (PMC12647871; doi:10.1038/s41598-025-25733-7)
Supplement: Supplementary file 1 — Supplementary Material 1 [file 41598_2025_25733_MOESM1_ESM.docx]

Supplementary information to

**Plasma Synthesis of Rhenium Nanoparticles as an Efficient Alternative to Platinum Nanoparticles for Nitroaromatic Compound Hydrogenations**

Piotr Cyganowski^1*^, Dominik Terefinko^2^, Mujahid Ameen Khan^2^, Agata Motyka-Pomagruk^3, 4^, Mateusz M. Marzec^5^, Sebastian Arabasz^6^, Krystian Sokolowski^5^, Pawel Pohl^2^, Andrzej Bernasik^5,7^, Aleksandra Goleniewska^2^, Piotr Jamroz^2^, Anna Dzimitrowicz^2*^

*^1^Department of Process Engineering and Technology of Polymer and Carbon Materials, Wroclaw University of Science and Technology, 27 Wybrzeze St. Wyspianskiego, 50-370 Wroclaw, Poland*

*^2^Department of Analytical Chemistry and Chemical Metallurgy, Wroclaw University of Science and Technology, 27 Wybrzeze St. Wyspianskiego, 50-370 Wroclaw, Poland*

*^3^ Laboratory of Plant Protection and Biotechnology, Intercollegiate Faculty of Biotechnology University of Gdansk and Medical University of Gdansk, University of Gdansk, 58 Abrahama, 80-307 Gdansk, Poland*

*^4^ Research & Development Laboratory, Intercollegiate Faculty of Biotechnology University of Gdansk and Medical University of Gdansk, University of Gdansk, 20 Podwale Przedmiejskie 20, 80-824 Gdansk, Poland*

*^5^Academic Centre for Materials and Nanotechnology, AGH University of Kraków, al. A. Mickiewicza 30, 30-059 Krakow, Poland*

*^6^Łukasiewicz Research Network - PORT Polish Center for Technology Development, 147 Stablowicka, 54-066 Wrocław, Poland*

*^7^Faculty of Physics and Applied Computer Science, AGH University of Kraków, 30 A. Mickiewicza Av., 30-059 Kraków, Poland*

*Corresponding Authors: prof. Piotr Cyganowski: Phone +48 58-523-52-08, e-mail address: [piotr.cyganowski@pwr.edu.pl](mailto:piotr.cyganowski@pwr.edu.pl) and prof. Anna Dzimitrowicz: Phone +48 71-320-24-53; e-mail address: [anna.dzimitrowicz@pwr.edu.pl](mailto:anna.dzimitrowicz@pwr.edu.pl)

**Table. S1.** Surface composition (atomic %) determined by fitting XPS data.

|  | **C** | | | | **N** | | **O** | | | **Si** | | | **Cl** | **Na** | **Re** | | | **Mg** | **Pt** | |
| --- | --- | --- | --- | --- | --- | --- | --- | --- | --- | --- | --- | --- | --- | --- | --- | --- | --- | --- | --- | --- |
| Binding energy [eV] | 285.0 | 286.6 | 288.3 | 288.9 | 400.2 | 402.0 | 531.7 | 532.8 | 533.9 | 98.9 | 101.8 | 103.2 | 198.8 | 1071.8 | 42.9 | 44.1 | 46.3 | 50.1 | 73.0 | 75.1 |
| Sample / Bonds | C-C | C-O, C-OH, C-N | C=O | O-C=O | C-NH | NH_4_^+^ | O-Re/O-Pt  O=C, O-S,O-Mg | O-C ,O-P, O-Si | -OH  H_2_O_ads_ | Si^0^ | silicone  siloxane | SiO_2_ | Cl^-^ | Na^+^ | Re^4+^ | Re^6+^ | Re^7+^ | Mg^2+^ | Pt^2+^ | Pt^4+^ |
| Re | 37.2 | 8.7 | 1.9 | 1.9 | 1.1 | 0.5 | 4.7 | 19.4 | 2.2 | 14.6 | 1.4 | 4.8 | 0.0 | 0.9 | 0.05 | 0.09 | 0.56 | - | - | - |
| Pt | 37.9 | 5.7 | 1.2 | 1.3 | 2.5 | 0.5 | 13.3 | 14.4 | 3.1 | 3.7 | 1.4 | 3.0 | 4.0 | 0.5 | - | - | - | - | 2.2 | 5.3 |

**
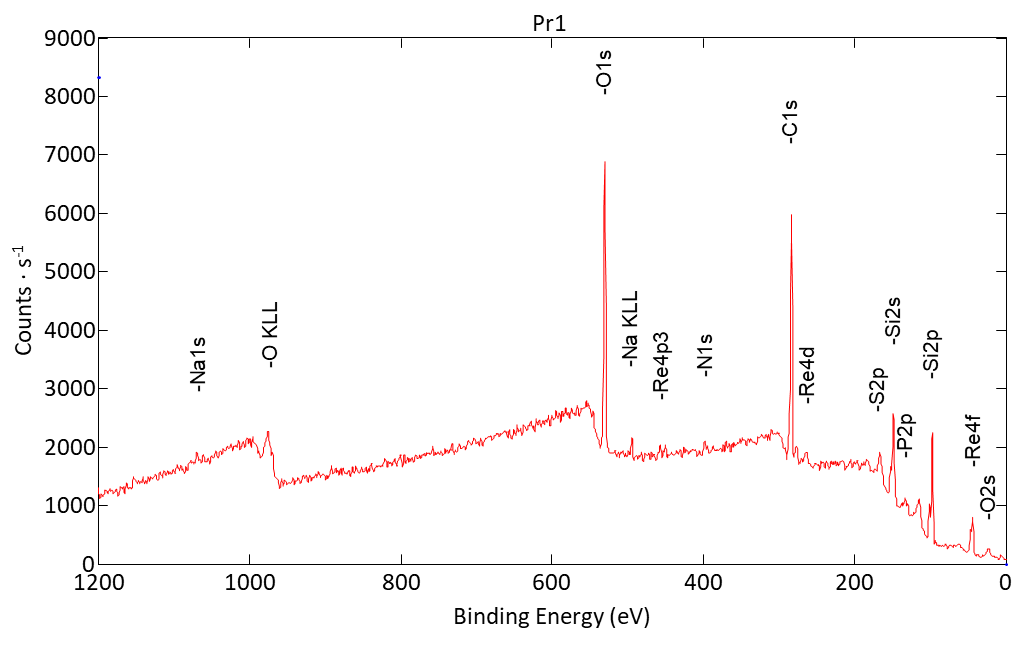
**

**Figure S1.** Survey XPS scan of ReNPs

**
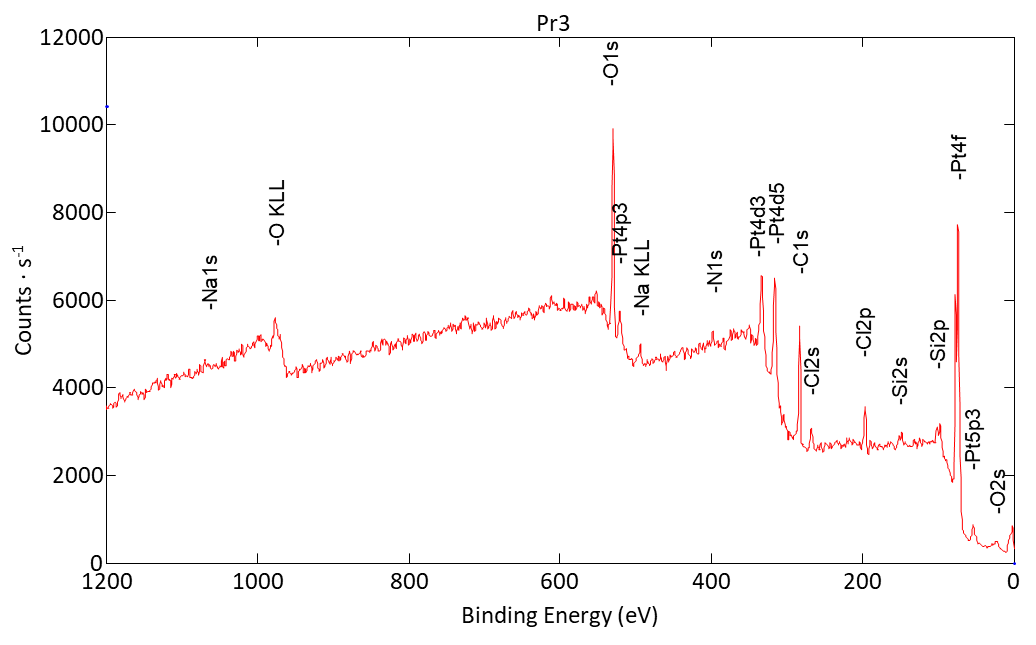
**

**Figure S2.** Survey XPS scan of PtNPs

**Figure S3.** High resolution XPS spectra of the sample containing ReNPs

**Figure S4.** High resolution XPS spectra of the sample containing PtNPs
